# Prognostic value of preoperative systemic immune-inflammation index in patients with bladder cancer undergoing radical cystectomy: a systematic review and meta-analysis

**DOI:** 10.3389/fonc.2026.1909255

**Published:** 2026-08-14

**Authors:** Maoliang Zhao, Yunxi Wu, Bing Luo, Jiacai Long, Jing Luo, Rong Liu, Heng Zhang, BingHan He

**Affiliations:** 1 Department of Urology, Bazhong Central Hospital, Bazhong, China; 2 Department of Pulmonary and Critical Care Medicine, Bazhong Central Hospital, Bazhong, China

**Keywords:** bladder cancer, meta-analysis, radical cystectomy, SII, systemic immune-inflammation index

## Abstract

**Background:**

To improve risk stratification in patients with bladder cancer (BC) undergoing radical cystectomy (RC), the systemic immune-inflammation index (SII), calculated from routine blood parameters, has attracted attention as a promising prognostic biomarker. This meta-analysis aims to synthesize existing evidence regarding the prognostic utility of SII and to elucidate its potential clinical significance.

**Methods:**

This systematic review and meta-analysis, conducted in accordance with the PRISMA framework, included nine cohort studies with a combined total of 6,288 participants. The prognostic impact of preoperative SII on postoperative outcomes was assessed by calculating pooled hazard ratios (HRs) using a random-effects model. Publication bias was assessed using funnel plots, Begg’s test, and Egger’s test, and its potential impact on the pooled estimates was critically evaluated.

**Results:**

The analysis revealed a significant correlation between elevated preoperative SII and adverse oncological outcomes. Notably, a higher SII was significantly associated with reduced overall survival (OS) (HR = 1.35, 95% CI: 1.15–1.59; p < 0.001). For recurrence-free survival (RFS), a potential association was observed (HR = 1.20, 95% CI: 1.01–1.41; p = 0.03); however, this finding was not robust in leave-one-out sensitivity analysis and should be considered highly tentative. For cancer-specific survival (CSS), a trend toward worse outcomes was noted (HR = 1.34, p = 0.05), suggesting a potential link that merits further investigation, however, this result was based on only three studies and should be interpreted with caution.

**Conclusions:**

The present study reveals that a high preoperative SII is associated with poorer OS in patients with BC undergoing RC, supporting its promise as a useful prognostic marker. However, these findings were markedly limited by substantial heterogeneity and clear evidence of publication bias (particularly for OS and RFS), suggesting that the true effect size may be smaller than estimated. To translate this promising finding into clinical practice, future large-scale, prospective multicenter studies are warranted. Such studies should focus on using standardized SII cut-offs and methodologies to validate its prognostic accuracy and suggest SII as a candidate prognostic marker worthy of further investigation. However, current evidence is insufficient to support its routine clinical use, particularly for RFS and CSS.

**Systematic review registration:**

https://www.crd.york.ac.uk/PROSPERO/, identifier CRD420251057070.

## 1 Introduction

Bladder cancer (BC) is one of the most common urological malignancies and represents a major global health burden. In 2022, roughly 614,000 new cases were diagnosed, and 220,000 deaths occurred due to the disease (1). Muscle-invasive bladder cancer (MIBC) constitutes the initial diagnosis in 30% of all BC cases. The remaining 70% of patients present with non-muscle-invasive bladder cancer (NMIBC), of whom approximately 21% eventually progress to MIBC. Despite multimodal therapy, outcomes remain heterogeneous (2). Patients with localized disease have a 60% 5-year survival rate, whereas this figure falls below 10% for individuals with distant metastatic disease (3). While radical cystectomy (RC) is the cornerstone of therapy for patients with MIBC and high-grade BC, long-term outcomes are often compromised by a high rate of tumor recurrence. Following surgery, as many as 70% of patients will face a relapse, manifesting as either local or systemic disease (4). The prognosis for patients experiencing pelvic recurrence is exceptionally poor, with median survival typically ranging from only 4 to 8 months, even with therapeutic intervention (5). Conventional prognostic models, which primarily rely on the Tumor Node Metastasis (TNM) staging system and pathological features, have limited accuracy in predicting individual patient outcomes. This limitation highlights the need for more reliable and accessible prognostic biomarkers to facilitate more precise patient stratification and therapeutic tailoring. Over the past decade, the complex interplay of systemic inflammatory responses and the advancement of cancer has garnered growing attention, with chronic inflammation now being established as a core hallmark of cancer (6). The systemic inflammatory response, mediated by a complex network of immune cells and cytokines, contributes to a pro-tumorigenic microenvironment that promotes tumor progression and adversely impacts patient survival (7). Consequently, various peripheral blood-based biomarkers reflecting the systemic inflammatory state have emerged as valuable prognostic tools in oncology (8).

Among these biomarkers, the neutrophil-to-lymphocyte ratio (NLR) and the platelet-to-lymphocyte ratio (PLR) have been widely investigated to stratify risk in patients with various solid tumors (9, 10). Emerging composite biomarkers such as the inflammatory burden index, pan-immune-inflammation value, and C-reactive protein-albumin-lymphocyte (CALLY) index have also been explored in bladder cancer cohorts, reflecting growing interest in immuno-nutritional profiling for risk stratification (11–13). More recently, the systemic immune-inflammation index (SII), a composite biomarker that integrates peripheral platelet, neutrophil, and lymphocyte counts as follows: (Platelet × Neutrophil)/Lymphocyte, has been proposed as a more comprehensive and potentially superior biomarker (14). It concurrently reflects the status of three crucial immune cell lineages: neutrophils, which can exert pro-tumor effects; lymphocytes, which represent the host’s primary anti-tumor immune response; and platelets, which are involved in tumor progression and angiogenesis. An elevated SII indicates an imbalanced inflammatory and immune status favorable to the tumor (15).

The prognostic significance of SII has been validated across a broad array of cancers, including lung (16), breast (17), and gastrointestinal cancers (18). In the context of bladder cancer, several individual studies have explored the association between preoperative SII and survival outcomes after treatment. However, these studies have often been limited by distinct therapeutic modalities and key differences in patient cohorts and analytical methods-such as variations in neoadjuvant/adjuvant therapy (19, 20) and pathological stages (21)-leading to inconsistent and sometimes conflicting results across different survival endpoints like overall survival (OS), recurrence-free survival (RFS), and cancer-specific survival (CSS) (22). This existing heterogeneity underscores the need for a quantitative synthesis of the available evidence.

In this study, we aimed to synthesize the current evidence to elucidate the predictive capacity of preoperative SII for OS, RFS, and CSS in patients treated with RC for BC.

## 2 Methods

### 2.1 Protocol

The protocol for this systematic review and meta-analysis was registered with PROSPERO (CRD420251057070. https://www.crd.york.ac.uk/PROSPERO/view/CRD420251057070), and the study was performed and reported following the Preferred Reporting Items for Systematic Reviews and Meta-Analyses (PRISMA) 2020 guidelines (23).

### 2.2 Literature search

A systematic literature search was conducted in PubMed, Web of Science, Embase, and the Cochrane Library for English-language publications, with no start date restriction up to May 19, 2025. The detailed search strategies for each database, including Boolean operators and filters, are provided in **Supplementary Table 1**. Only English-language publications were included due to resource constraints for translation. Furthermore, forward and backward citation searches (snowballing) of all included articles and key reviews were conducted to ensure the comprehensive identification of all potentially relevant studies.

### 2.3 Inclusion and exclusion criteria

Eligible studies enrolled patients with bladder cancer (BC) undergoing radical cystectomy (RC) and categorized participants according to preoperative systemic immune-inflammation index (SII) levels. Studies were required to assess the association between preoperative SII and oncological outcomes, including overall survival (OS), recurrence-free survival (RFS), and/or cancer-specific survival (CSS), and to report hazard ratios (HRs) with 95% confidence intervals (CIs) or provide sufficient data for their estimation. Both prospective and retrospective cohort studies were considered eligible.

Exclusion criteria: Studies not employing an original cohort design were deemed ineligible for inclusion (e.g., reviews, editorials, preclinical studies) or if they were cross-sectional. Additionally, studies lacking preoperative SII data or the necessary survival outcomes were excluded. In instances of duplicate cohorts, the study reporting on the largest patient cohort was prioritized for inclusion in the definitive analysis.

OS was defined as the time from the date of surgery or diagnosis to death from any cause. CSS was defined as the time from the date of surgery or diagnosis to death due to bladder cancer. RFS was defined as the time from the date of surgery or diagnosis to disease recurrence (local or distant) or death from any cause.

### 2.4 Data extraction and quality assessment

The data extraction process was conducted independently by two reviewers. Any conflicts were addressed via discussion, with a third reviewer mediating unresolved discrepancies. The extracted dataset encompassed study characteristics (first author, year, location, design), patient demographics (sample size, age), clinical information (tumor type, treatment modality), details on the SII biomarker (cut-off value and determination method), and survival outcomes (follow-up duration, OS, CSS, RFS). All survival outcomes were extracted as HR with corresponding 95% CI. When studies presented both unadjusted and adjusted statistical results, the latter were preferentially selected to obtain more robust effect estimates. The specific covariates included in each multivariable model are summarized in **Supplementary Table 2**. In cases of incomplete data or unclear methodology, attempts were made to contact the corresponding authors to obtain additional information. Methodological appraisal was conducted for all included studies via the Newcastle–Ottawa Scale (24). Studies achieving a score of 7 points or greater on the scale were categorized as high-quality.

### 2.5 Statistical analysis

The principal effect measure was the summary Hazard Ratio (HR) with its 95% CI, employed to evaluate the prognostic impact of preoperative SII on survival outcomes. Between-study heterogeneity was assessed via the I² value. and its statistical significance was determined by the Chi-squared based Q-test. A finding of I² > 50% or a p-value < 0.10 was considered to represent considerable heterogeneity (25). Consequently, all effect estimates were aggregated under a random-effects model that accounts for between-study variance.

To identify potential sources of heterogeneity, predefined subgroup analyses were performed based on factors such as region, sample size, age, and SII cutoff values. To explore the impact of SII cutoff values on the pooled effect estimates, we performed a *post-hoc* subgroup analysis based on the median cutoff value (600) across the included studies, categorizing studies into low cutoff (<600) and high cutoff (≥600) groups. To assess the stability of our results, we conducted a leave-one-out sensitivity analysis, in which each study was systematically removed to observe its impact on the pooled estimate. Furthermore, we investigated potential publication bias by visually inspecting the funnel plot for asymmetry and by performing both Begg’s (26) and Egger’s (27) statistical tests. Statistical analysis was carried out with Stata software (Version 18.0; StataCorp. College Station, TX, USA). For all analyses, statistical significance was defined as a p-value < 0.05.

To further explore potential sources of heterogeneity, we performed univariate meta-regression analyses using the restricted maximum likelihood (REML) method to evaluate whether study-level covariates, including SII cutoff value, median age, and sample size, were associated with the log-transformed HR for OS. A random-effects model with restricted maximum likelihood estimation was fitted for each covariate. The p-value < 0.05 was considered statistically significant.

## 3 Results

### 3.1 Study selection

**Figure 1:**
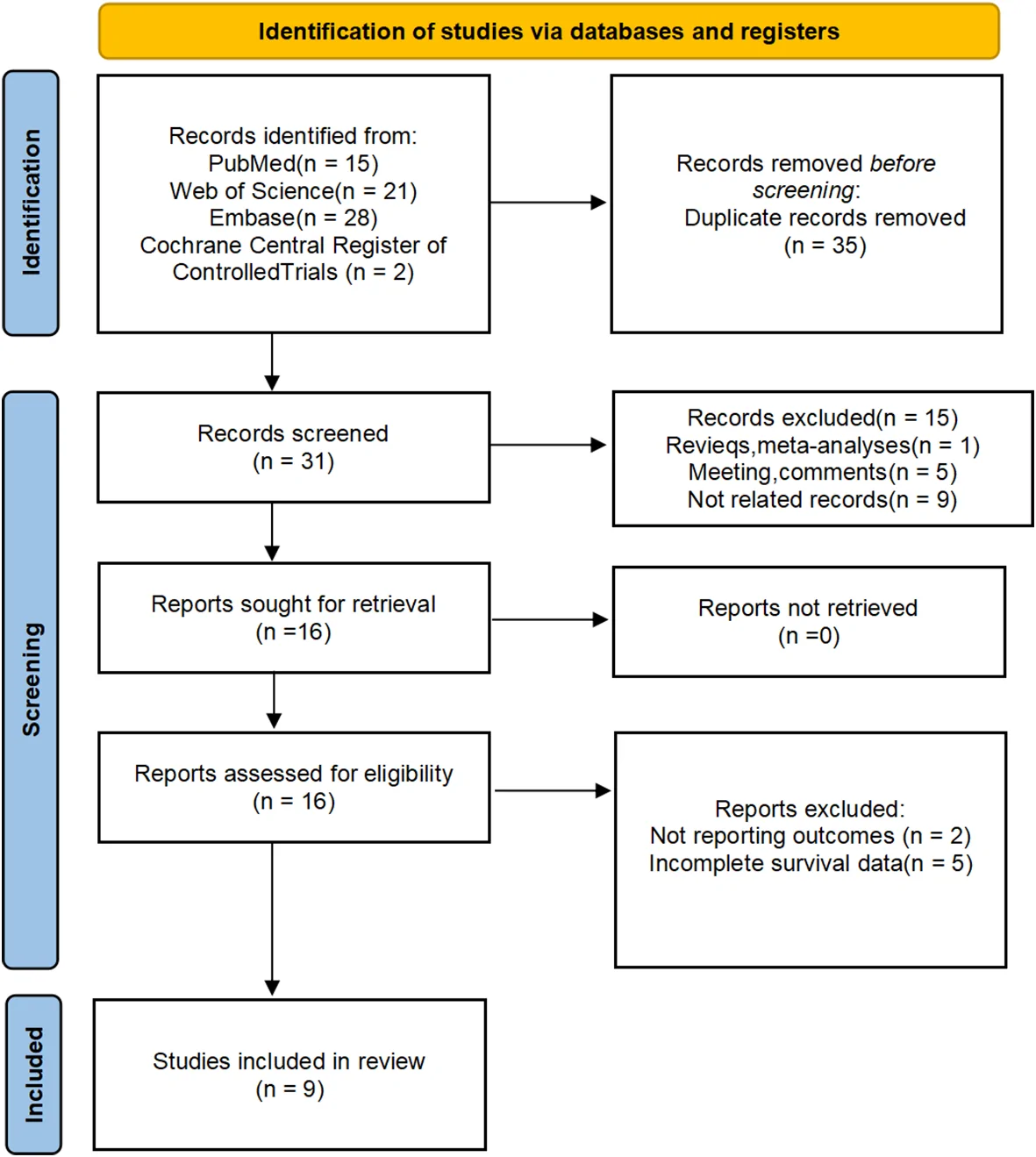
Flow diagram of the study selection process.

A total of 66 records were initially identified through the literature search. Following the removal of 35 duplicates, 31 records underwent title and abstract screening, leading to the exclusion of 15 studies. The 16 articles that advanced to the next stage were then subjected to a comprehensive full-text evaluation to determine their eligibility. Following this in-depth review, a further 7 publications were eliminated as they did not conform to the pre-defined selection standards. Ultimately, the final quantitative synthesis was based on 9 publications (28–36), collectively encompassing 6,288 patients, all of which were published between 2019 and 2024. The PRISMA-compliant study selection and screening process is depicted schematically in **Figure 1**.

### 3.2 Study characteristics

The final set of nine publications originated from diverse geographical locations: multicountry (n=1) (28), China (n = 3) (29, 33, 34)), Italy (n = 1) (30), Turkey (n = 2) (31, 36), Iran (n = 1) (32), and Japan (n = 1) (35). All of these articles utilized a retrospective methodology. The number of patients per study varied, with individual cohorts comprising between 79 and 4,335 individuals. Median baseline age varied from 60.8 to 78 years and follow-up durations ranged from 14.7 to 42 months. The thresholds used to define an elevated SII varied across studies, spanning from 438 to 1228. OS was reported in all nine studies (28–36), RFS in five (28–30, 33, 36), and CSS in three (28, 30, 35). Methodological quality was high across all studies, with Newcastle–Ottawa Scale (NOS) scores ≥7. A detailed overview of the included studies and their baseline characteristics is provided in **Table 1**.

**Table 1:** Baseline characteristics of included studies and quality evaluation.

Author	Study period	Country	Study design	Sample size	Mean/ Median age(year)	Male	SII cutoff	Tumor type	Clinical tumor stage	Survival analysis	Median follow-up (months)	NOS score	Perioperative therapy reported
Grossmann 2022 (28)	NR	Multicountry	Retrospective	4,335	67 (median)	3,464	610	BC	Ta-T4	OS, RFS, CSS	42	8	AC: 23%; Excluded NAC
Tang 2020 (29)	2014 -2020	China	Retrospective	79	63.62 (mean)	70	OS547.30 / RFS778	BC	Ta-T4	OS, RFS	31	7	NR
Russo 2023 (30)	2016 -2022	Italy	Retrospective	193	78 (median)	154	640.27	BC	Ta-T4	OS, RFS, CSS	20	8	AC: 20.7%
Yilmaz 2020 (31)	1999 -2019	Turkey	Retrospective	152	66 (median)	133	768	MIBC	T2-T4	OS	16	7	NAC: 28.2%
Salari 2024 (32)	2016 -2022	Iran	Retrospective	187	60.8 (mean)	179	410.66	MIBC	NR	OS	14.7	8	NAC: 50.3%
Zhang 2022 (33)	2010 -2020	China	Retrospective	725	65 (median)	621	554.23	BC	Ta-T4	OS, RFS	36	8	AC in 14.71% NAC in 6.09%
Zhang 2019 (34)	2005 -2019	China	Retrospective	139	67 (median)	121	507	MIBC	Tis-T4	OS	NR	8	AC: 23.7%
Yamashita 2021 (35)	2009 -2018	Japan	Retrospective	237	73 (median)	188	438	BC	Ta-T4	OS, CSS	38	8	NAC: 30%
Yilmaz 2023 (36)	2009 -2021	Turkey	Retrospective	241	65 (median)	205	1228	BC	T1-T4	OS, RFS	20	8	NAC: 17%

AC, adjuvant chemotherapy; BC, bladder cancer; CSS, cancer-specific survival; MIBC, muscle-invasive bladder cancer; NAC, neoadjuvant chemotherapy; NMIBC, non-muscle-invasive bladder cancer; NR, not reported; OS, overall survival; RFS, recurrence-free survival.

Regarding perioperative therapy, eight studies reported the use of neoadjuvant or adjuvant chemotherapy, with NAC utilization rates ranging from 17% to 50.3% and adjuvant chemotherapy rates ranging from 14.71% to 23.7%; and one study (Tang et al.) did not report any perioperative treatment information. The methods used to determine SII cutoffs also varied: seven studies employed ROC analysis with the Youden index, while two studies used X-tile software (**Supplementary Table 3**).

### 3.3 OS

Nine studies with a combined total of 6,288 patients undergoing RC for BC, provided data on the prognostic role of preoperative SII for OS (28–36). High preoperative SII was significantly associated with unfavorable OS (HR = 1.35; 95% CI: 1.15–1.59; p < 0.001) with substantial heterogeneity among studies (I² = 82.8%; p < 0.001) (**Supplementary Figure 1A**; **Table 2**). To further dissect the origins of the between-study variance, we conducted additional subgroup analyses based on several key factors: geographic region, sample size, median patient age, and SII cut-off value. In this study, the median SII cutoff value (600) from the nine included studies was used as the threshold for subgroup analysis. A significant link between high SII and unfavorable OS was observed in the Mediterranean-West Asia group, among patients aged >65 years, and regardless of sample size or SII cutoff value (all p < 0.05). However, our analysis failed to demonstrate a statistically significant relationship in the East Asian group (p = 0.060) or among patients aged ≤65 years (p = 0.06) (**Table 2**).

**Table 2:** Subgroup analyses of OS, RFS, and CSS.

Outcome	Variable	No. of studies	HR (95% CI)	P value	Heterogeneity	
					I² (%)	P value
OS	All	9	1.35 (1.15, 1.59)	< 0.001	82.80	< 0.001
Region	East Asia	4	1.36 (0.99, 1.88)	0.060	83.00	0.001
	Mediterranean-West Asia	5	1.54 (1.11, 2.12)	0.009	69.20	0.011
Sample size	> 200	4	1.41 (1.08, 1.85)	0.012	77.70	0.004
	< 200	5	1.55 (1.01, 2.36)	0.044	76.70	0.002
Age	> 65	5	1.59 (1.11, 2.28)	0.011	71.90	0.007
	≤ 65	4	1.33 (0.99, 1.80)	0.061	82.30	< 0.001
Cutoff	≥ 600	4	1.45 (1.04, 2.03)	0.029	69.80	0.019
	< 600	5	1.46 (1.06, 2.02)	0.019	82.30	< 0.001
RFS	All	5	1.20 (1.01, 1.41)	0.033	81.40	< 0.001
Region	East Asia	2	1.04 (0.91, 1.20)	0.552	50.40	0.155
	Mediterranean-West Asia	3	1.63 (0.98, 2.73)	0.061	79.10	0.008
Sample size	≥ 200	3	1.25 (1.00, 1.56)	0.053	61.20	0.076
	< 200	2	1.38 (0.67, 2.84)	0.384	85.90	0.008
Age	> 65	2	1.45 (0.80, 2.65)	0.222	79.10	0.029
	≤ 65	3	1.21 (0.91, 1.60)	0.187	78.90	0.009
Cutoff	≥ 600	4	1.21 (1.00, 1.47)	0.052	84.60	< 0.001
	< 600	1	1.18 (0.94, 1.48)	0.153	0.00	< 0.001
CSS	All	3	1.34 (1.00, 1.81)	0.052	42.40	0.176
Region	East Asia	1	1.97 (1.16, 3.36)	0.013	0.00	< 0.001
	Mediterranean-West Asia	2	1.18 (1.05, 1.33)	0.004	0.00	0.715
Sample size	≥ 200	2	1.42 (0.88, 2.31)	0.153	70.50	0.066
	< 200	1	1.33 (0.71, 2.50)	0.376	0.00	< 0.001
Cutoff	≥ 600	2	1.18 (1.05, 1.33)	0.004	0.00	0.715
	< 600	1	1.97 (1.16, 3.36)	0.013	0.00	< 0.001

CI, confidence interval; CSS, cancer-specific survival; HR, hazard ratio; OS, overall survival; RFS, recurrence-free survival.

To investigate potential sources of heterogeneity in the pooled OS analysis, random-effects meta-regression analyses were performed. Study-level variables including SII cutoff values, median age, and sample size were evaluated as potential moderators. None of these factors significantly accounted for the observed heterogeneity (SII cutoff: β = −0.00082, p=0.124; median age: β = 0.0159, p=0.606; sample size: β = −0.000108, p=0.281) (**Supplementary Table 4**; **Supplementary Figure 2**). These findings suggest that the heterogeneity may be attributable to other unmeasured clinical or methodological differences among studies.

### 3.4 RFS

The prognostic value of preoperative SII for RFS was evaluated in a pooled analysis of five studies, encompassing a total cohort of 5,573 patients undergoing RC for BC (28–30, 33, 36). A high preoperative SII level was independently associated with a 20% increased risk of RFS events (summary HR = 1.20; 95% CI: 1.01–1.41; p = 0.03). This finding should be interpreted with caution, given the high level of variability observed between the contributing studies (I² = 81.4%; p < 0.001) (**Supplementary Figure 1B**; **Table 2**). Subgroup analyses were further conducted based on geographic region, sample size, median patient age, and SII cut-off value. No statistically significant association was observed across any of the subgroups (all p > 0.05) (**Table 2**).

### 3.5 CSS

The prognostic significance of preoperative SII for Cancer-Specific Survival (CSS) was addressed by three investigations, covering a combined total of 5,765 individuals treated with RC for BC (28, 30, 35). The pooled analysis indicated a trend towards worse CSS in patients with high preoperative SII, which was of borderline statistical significant (HR = 1.34; 95% CI: 1.00–1.81; p = 0.05), with moderate heterogeneity among studies (I² = 42.4%; p = 0.18) (**Supplementary Figure 1C**; **Table 2**). Only three studies were available for analysis. With such a small number of studies, both Begg’s test and Egger’s test lack statistical power to detect publication bias, and the funnel plot cannot be reliably interpreted for asymmetry. Therefore, this finding should be interpreted with caution. Subgroup analyses were further conducted based on geographic region, sample size, and SII cutoff values. A significant link between high SII and unfavorable CSS was observed regardless of region or SII cutoff value (all p < 0.05). By contrast, no statistically significant association was found in subgroups stratified by sample size (all p > 0.05).

### 3.6 Sensitivity analyses

To evaluate the stability of the summary HR for each outcome, we performed a leave-one-out analysis. For OS, the prognostic link between a high preoperative SII and adverse outcomes remained stable, showing no significant alteration upon the systematic removal of any single study, indicating a robust and stable result (**Supplementary Figure 3A**).

For RFS, the statistical significance of the association was sensitive to the inclusion of the study by Grossmann et al. (28) suggesting potential instability (**Supplementary Figure 3B**).

Regarding CSS, the limited number of available studies (n = 3) resulted in unstable pooled estimates, with loss of significance upon omission of any single study (**Supplementary Figure 3C**).

These findings indicate that the results for OS were robust, whereas the associations for RFS and CSS were less stable and require cautious interpretation.

### 3.7 Publication bias

A multi-faceted approach was utilized to evaluate publication bias for each survival outcome, incorporating graphical analysis of funnel plots alongside the quantitative methods of Begg’s and Egger’s tests.

For OS (9 studies), the funnel plot appeared asymmetric (**Supplementary Figure 4A**). While Begg’s test was non-significant (p = 0.84), Egger’s test detected significant small-study effects (p < 0.001), indicating possible publication bias.

For RFS (5 studies), a similar asymmetry was noted (**Supplementary Figure 4B**). The statistical tests for publication bias offered convergent evidence of asymmetry; Begg’s test reached the threshold of significance (p = 0.05), while Egger’s test was unequivocally significant (p = 0.005).

For CSS (3 studies), the limited number of studies (n = 3) restricted the power to detect publication bias. Consequently, while the funnel plot is presented for review (**Supplementary Figure 4C**), both the visual interpretation and the statistical results from Begg’s (p = 0.60) and Egger’s (p = 0.40) tests were considered uninformative.

## 4 Discussion

Survival outcomes are markedly heterogeneous for BC patients after RC. Therefore, there is a pressing clinical requirement for readily accessible, cost-effective biomarkers to aid in developing treatment plans, detecting relapses, and improving preoperative risk stratification. The balance of the host’s pro-inflammatory and immune surveillance functions is a key determinant in the pathogenesis of carcinoma. SII offers a simple method to gauge this status by combining neutrophil, platelet, and lymphocyte counts into a single value. This integrated index quantitatively reflects a systemic inflammatory state that is strongly associated with cancer progression (37) and may hold an advantage over other markers in predicting prognosis for inflammation-related conditions.

Mechanistically, a high SII value represents a systemic immune balance shifted from effective anti-tumor surveillance toward a pro-tumorigenic inflammatory state that fuels cancer progression. An elevated neutrophil count signifies a state of tumor-promoting inflammation, as recruited neutrophils shape the tumor microenvironment by releasing reactive oxygen species (ROS) to induce genetic mutations and secreting enzymes that facilitate invasion and metastasis. Conversely, a low lymphocyte count indicates that the host’s adaptive anti-tumor immunity is impaired, reflecting the tumor’s success in evading immune destruction-a crucial hallmark capability that allows for unchecked growth and is linked to poor prognosis (38, 39). The existing literature supports SII’s value as a non-invasive tool for prognostic risk assessment in urologic oncology (40), predicting worse survival outcomes in urothelial carcinoma (41), and predicting unfavorable prognoses for both patients with renal cell carcinoma (42) and individuals who have completed radical prostatectomy (43).

The present investigation synthesizes existing literature via a systematic review and meta-analysis to assess the utility of the preoperative SII as a prognostic marker in the specific cohort of patients undergoing RC for BC. The most compelling conclusion from our analysis is that a high preoperative SII serves as a strong predictor of unfavorable OS. The stability of this association across sensitivity analyses reinforces the potential of the SII for preoperative risk stratification, particularly given its affordability and ease of measurement.

Our findings align with a broader body of literature evaluating systemic inflammation-based biomarkers in MIBC. Recent studies have reported that the inflammatory burden index, pan-immune-inflammation value, and CALLY index are also associated with survival outcomes in bladder cancer patients treated with RC or other modalities (11–13). This collective evidence suggests that composite indices integrating multiple hematological parameters may offer improved prognostic information over single markers. However, similar to SII, these novel indices face challenges related to cut-off standardization and validation across diverse populations.

In contrast, the evidence for RFS and CSS is less conclusive and highlights a more complex landscape. For RFS, although our initial pooled analysis indicated a statistically significant association, the finding lacked robustness. The statistical significance appears largely driven by a single study (28) and is further undermined by unequivocal publication bias (Egger’s test p = 0.005). In addition, both visual and quantitative methods raised concerns regarding the potential for skewed reporting in the literature. Therefore, based on the current body of evidence, our analysis does not provide robust evidence for SII as a predictor of RFS. This discrepancy suggests that while a prognostic signal for recurrence exists—as SII has demonstrated independent prognostic value for disease recurrence in other distinct cohorts, such as NMIBC patients undergoing immunotherapy (44)—its consistency may be affected by factors such as surgical quality, adjuvant or neoadjuvant therapy (19, 20) and key pathological features like lymph node density (21).

The relationship between SII and CSS is even more tenuous. With only three studies (28, 30, 35) available for meta-analysis, the pooled result was not statistically significant (p = 0.05) and proved highly unstable in sensitivity analysis. Given the sparse data and lack of statistical power, current evidence is insufficient to determine the prognostic value of SII for CSS. We therefore caution against overinterpreting the result as definitive evidence, although the biological signal linking SII to cancer-specific mortality may still be present and warrants further investigation with larger, more homogeneous study populations.

Among the included studies, NAC utilization rates varied substantially, ranging from 17% to 50.3% in those that reported this information, while one study did not report any perioperative treatment data. This considerable variability, combined with the lack of uniform reporting, prevented us from performing a subgroup analysis stratified by NAC exposure. Similarly, variations in adjuvant chemotherapy regimens may have influenced postoperative survival outcomes, further contributing to the observed heterogeneity. These treatment-related confounders represent a significant limitation of the current evidence base and underscore the need for future studies to consistently report perioperative treatment details, ideally with sufficient granularity to allow for stratified analyses. Recent years have witnessed a growing interest in optimizing perioperative management and prognostic stratification for bladder cancer patients undergoing radical cystectomy, with recent studies extending beyond SII to examine both surgical outcomes and broader prognostic biomarkers (45, 46).

Interpretation of these findings is constrained by several key limitations, including heterogeneity and publication bias. Publication bias is a particular concern. The asymmetric funnel plots for OS and RFS, supported by significant Egger’s test results (p < 0.001 and p = 0.005, respectively), strongly indicate the presence of small-study effects. This observation is consistent with the “file drawer problem”, a form of publication bias where smaller studies are more likely to reach publication if they report statistically significant positive results, while those with non-significant or negative findings tend to go unpublished (47). In the context of prognostic biomarker meta-analyses, smaller studies—often more susceptible to methodological heterogeneity, selective outcome reporting, and inadequate adjustment for confounding—are particularly prone to producing inflated effect estimates. Consequently, the observed HRs should be viewed as provisional upper-bound estimates rather than definitive measures of the true prognostic effect, which is likely more modest. This potential overestimation warrants significant caution when interpreting the clinical relevance of our findings, and for CSS, the small number (n=3) of studies precluded any meaningful assessment of bias.

Furthermore, the substantial heterogeneity observed for OS (I² = 82.8%) and RFS (I² = 81.4%) requires careful consideration as it complicates the direct interpretation of the pooled estimates. This heterogeneity likely stems from several key sources. First and foremost is the lack of a standardized SII cutoff value, which ranged widely from 438 to 1228 across the included studies. The various methods used to determine these cutoffs, such as receiver operating characteristic (ROC) curve analysis or using the median value, result in different patient stratifications and may directly contribute to the variability in reported hazard ratios. However, our meta-regression analyses did not identify any of the examined study-level covariates as significant moderators of the pooled OS effect. This suggests that the observed heterogeneity is unlikely to be attributable to a single, easily measurable factor. More likely, it reflects a confluence of unmeasured confounders, including differences in perioperative treatment protocols, surgical techniques, patient selection criteria, and pathological assessment standards across institutions. In addition, significant geographic differences were noted in our subgroup analysis, with no significant association found for OS in the East Asian group. This raises the question of whether this is a statistical artifact of fewer studies or reflects true underlying differences in baseline inflammatory status, genetics, or lifestyle among populations. Finally, the inconsistent application of neoadjuvant and adjuvant therapies across the patient cohorts is a major unmeasured confounder. Since these treatments profoundly impact survival outcomes, variations in their use could have significantly influenced the reported prognostic value of SII, contributing to the observed heterogeneity. This underscores the need for more standardized reporting in future studies to enable more robust identification of heterogeneity sources.

The covariates adjusted for in the multivariable models varied considerably across the included studies, which may affect the comparability of the extracted HRs and contribute to the observed heterogeneity. This limitation underscores the need for standardized reporting of prognostic factor analyses in future studies. And then, limiting the literature to English may introduce language bias, as relevant non-English studies may be missed. This is a common limitation in meta-analyses.

These limitations, along with the retrospective design of all included studies, underscore the preliminary nature of the evidence, especially for RFS and CSS.

## 5 Conclusion

The evidence synthesized in this review indicates that high preoperative SII levels are associated with poorer overall prognosis in bladder cancer patients undergoing radical cystectomy. However, owing to substantial heterogeneity and clear evidence of publication bias that may have overestimated the true effect, this finding should be interpreted with caution. For RFS, the observed association was not robust to sensitivity analysis, and for CSS, the evidence was insufficient due to the limited number of studies.

Therefore, while SII remains a promising research biomarker, its readiness for clinical application is not supported by the current evidence. Future large-scale, prospective multicenter studies with standardized methodologies, particularly uniform SII cutoffs and consistent reporting of therapeutic regimens, are imperative to validate these findings and overcome the common challenges of biomarker development, enabling the reliable integration of SII into clinical practice (48).

## Data Availability

The datasets presented in this study can be found in online repositories. The names of the repository/repositories and accession number(s) can be found in the article/**Supplementary Material**.
