# Supplementary material for: Prognostic value of preoperative systemic immune-inflammation index in patients with bladder cancer undergoing radical cystectomy: a systematic review and meta-analysis
Source: Front Oncol. 2026 Aug 14;16:1909255. doi: 10.3389/fonc.2026.1909255 (PMC13521783; doi:10.3389/fonc.2026.1909255)
Supplement: Supplementary file 1 [file DataSheet1.pdf]

Supplementary Material

1 Supplementary Figures

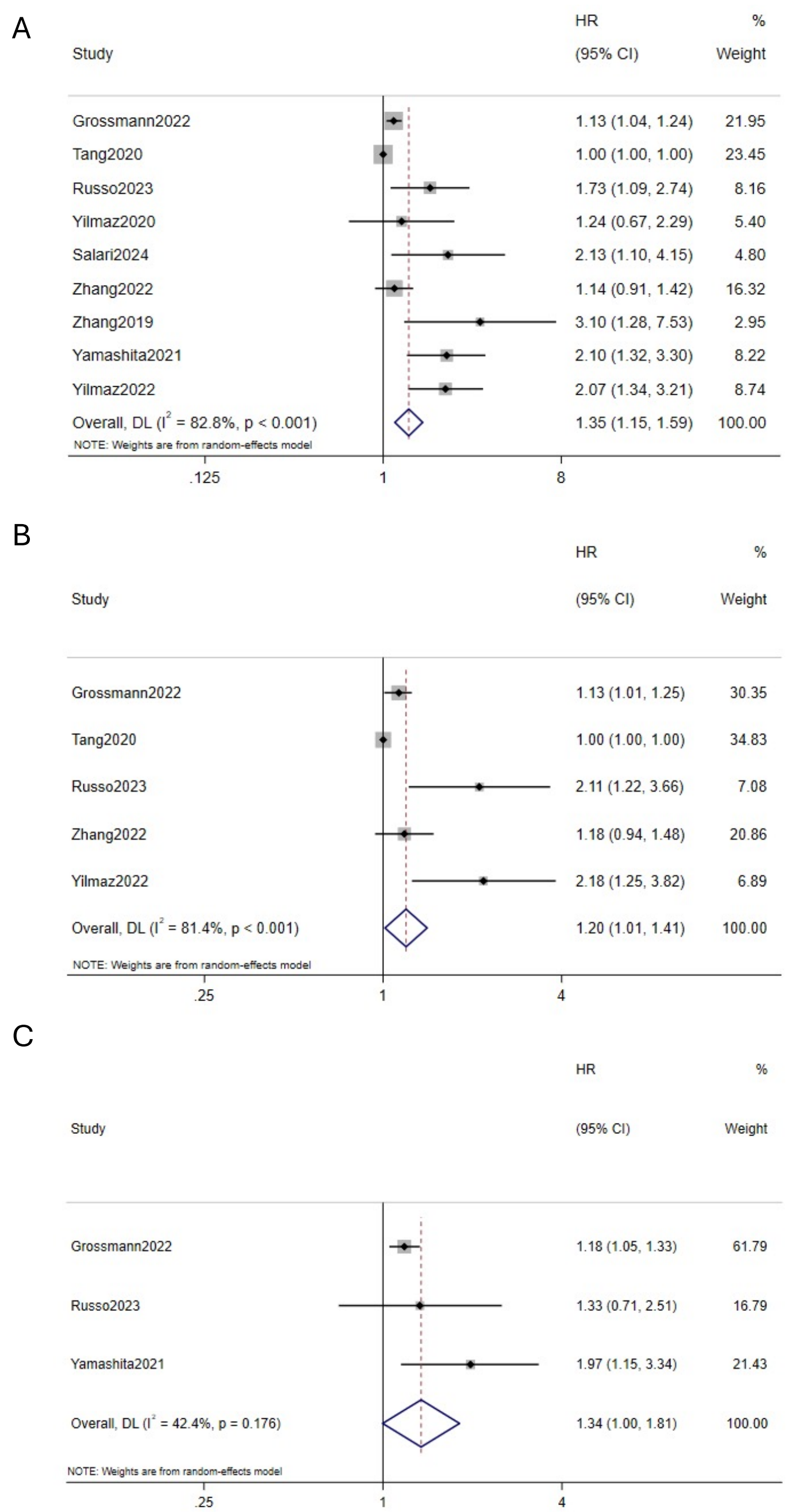

Supplementary Figure 1. Forest plot of the (A) OS, (B) RFS, (C) CSS. OS, overall survival; RFS, recurrence-free survival; CSS, cancer-specific survival.

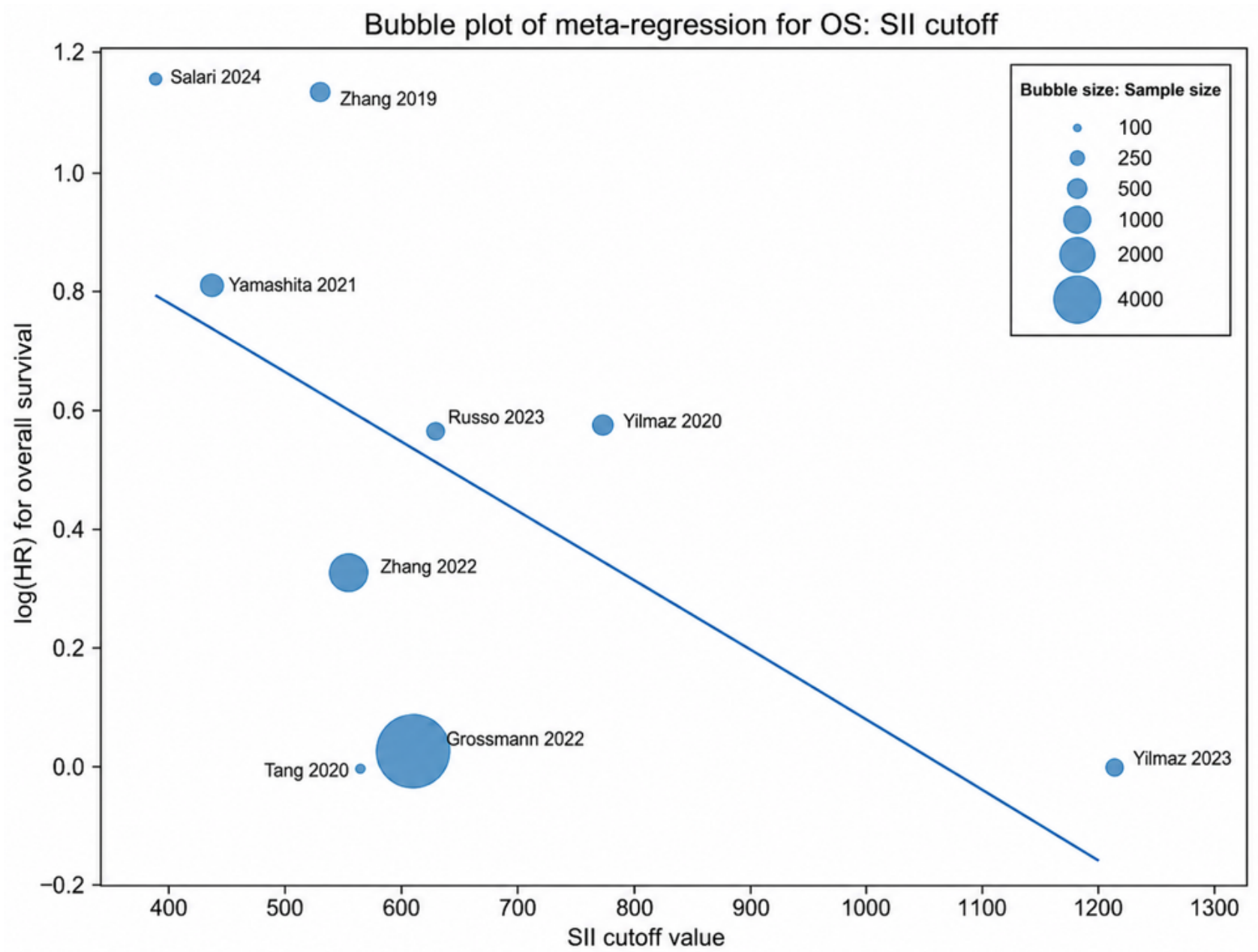

Supplementary Figure 2. Bubble plot of the meta-regression analysis examining the association between SII cutoff values and overall survival (OS) effect estimates. Each bubble represents an individual study. The x-axis indicates the SII cutoff value used in each study, and the y-axis represents the log-transformed hazard ratio (log HR) for OS. The size of each bubble is proportional to the study's sample size, with larger bubbles indicating larger samples. The solid regression line represents the predicted log HR across SII cutoff values.

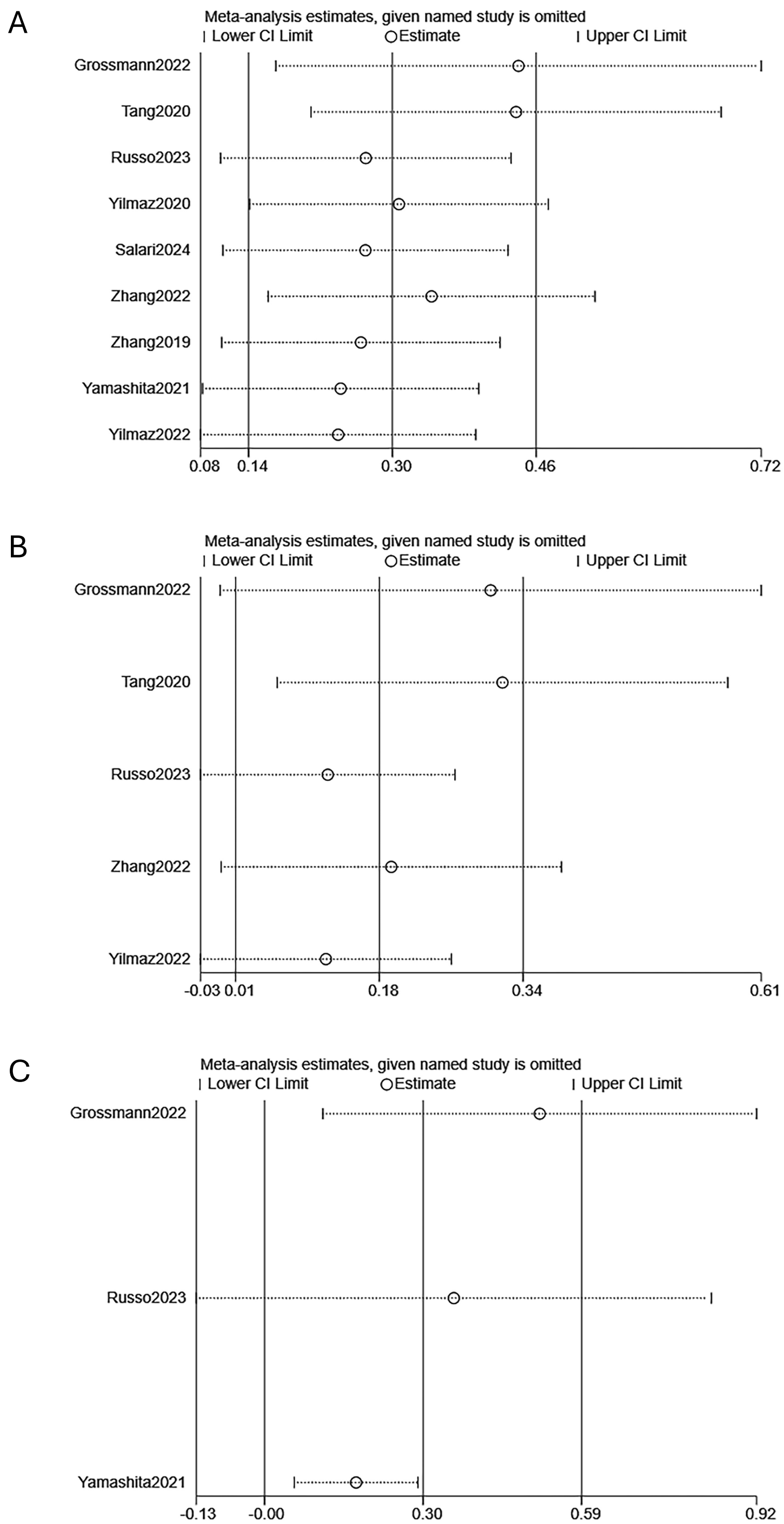

Supplementary Figure 3. Leave-one-out sensitivity analysis of the (A) OS, (B) RFS, (C) CSS. OS, overall survival; RFS, recurrence-free survival; CSS, cancer-specific survival.

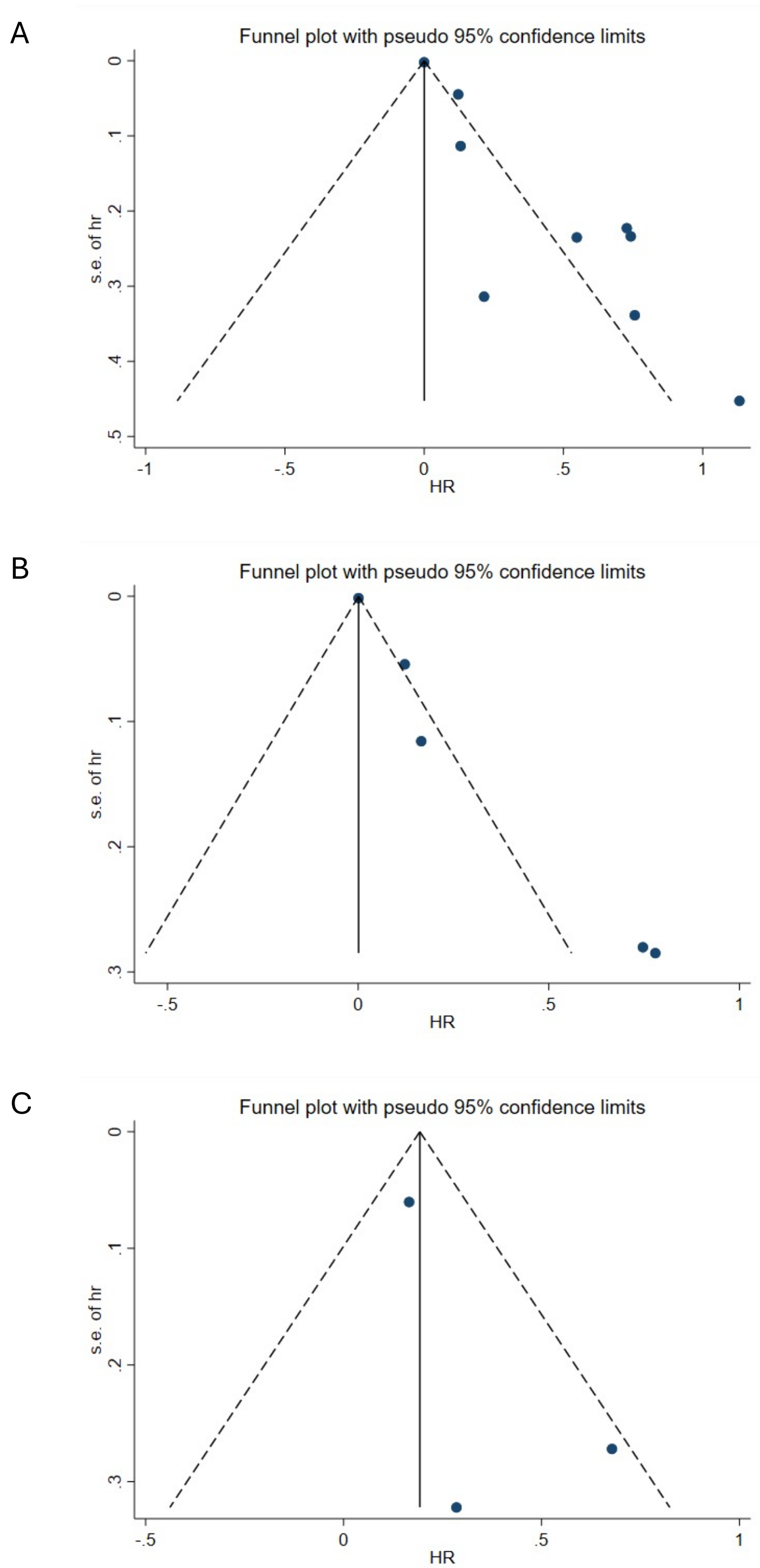

Supplementary Figure 4. Begg's funnel plot for publication bias assessment of the (A) OS, (B) RFS, (C) CSS. OS, overall survival; RFS, recurrence-free survival; CSS, cancer-specific survival.
