# Supplementary material for: Prognostic value of preoperative systemic immune-inflammation index in patients with bladder cancer undergoing radical cystectomy: a systematic review and meta-analysis
Source: Front Oncol. 2026 Aug 14;16:1909255. doi: 10.3389/fonc.2026.1909255 (PMC13521783; doi:10.3389/fonc.2026.1909255)
Supplement: Supplementary file 2 [file Table1.docx]

**Supplementary Table 1 |** Complete search strategies for each database

| Database | Search Strategy | Results |
| --- | --- | --- |
| PubMed | ("cystectomy"[MeSH Terms] OR ("cystectomy"[MeSH Terms] OR "cystectomy"[All Fields] OR "cystectomies"[All Fields] OR ("cystectomy"[MeSH Terms] OR "cystectomy"[All Fields] OR ("radical"[All Fields] AND "cystectomy"[All Fields]) OR "radical cystectomy"[All Fields]) OR ("cystectomy"[MeSH Terms] OR "cystectomy"[All Fields] OR ("Cystectomy"[All Fields] AND "radical"[All Fields]) OR "cystectomy radical"[All Fields]) OR ("cystectomy"[MeSH Terms] OR "cystectomy"[All Fields] OR ("radical"[All Fields] AND "cystectomies"[All Fields]) OR "radical cystectomies"[All Fields]))) AND ((("systemic"[All Fields] OR "systemically"[All Fields] OR "systemics"[All Fields]) AND "immune-inflammation"[All Fields] AND ("abstracting and indexing"[MeSH Terms] OR ("abstracting"[All Fields] AND "indexing"[All Fields]) OR "abstracting and indexing"[All Fields] OR "index"[All Fields] OR "indexed"[All Fields] OR "indexes"[All Fields] OR "indexing"[All Fields] OR "indexation"[All Fields] OR "indexations"[All Fields] OR "indexe"[All Fields] OR "indexer"[All Fields] OR "indexers"[All Fields] OR "indexs"[All Fields])) OR ("stat interface"[Journal] OR "sii"[All Fields])) | 15 |
| Embase | (exp cystectomy OR cystectomy.ti,ab,kf.) AND ((systemic immune-inflammation index OR systemic immune inflammation index OR systemic immune-inflammatory index OR systemic immunoinflammatory index OR SII).ti,ab,kf.) | 28 |
| Cochrane Library | ([mh cystectomy] OR cystectom*:ti,ab,kw) AND ("systemic immune-inflammation index":ti,ab,kw OR "systemic immune inflammation index":ti,ab,kw OR "systemic immune-inflammatory index":ti,ab,kw OR "systemic immunoinflammatory index":ti,ab,kw OR SII:ti,ab,kw) | 2 |
| Web of Science | TS=(cystectom* AND ("systemic immune-inflammation index" OR "systemic immune inflammation index" OR "systemic immune-inflammatory index" OR "systemic immunoinflammatory index" OR SII)) | 21 |

Abbreviations: MeSH, Medical Subject Headings; TS, topic search; ti, title; ab, abstract; kf, keyword heading; kw, keyword; exp, exploded search. All searches were limited to English-language publications with no restriction on publication date. The final search was performed on May 19, 2025.
