# Supplementary material for: Prognostic value of preoperative systemic immune-inflammation index in patients with bladder cancer undergoing radical cystectomy: a systematic review and meta-analysis
Source: Front Oncol. 2026 Aug 14;16:1909255. doi: 10.3389/fonc.2026.1909255 (PMC13521783; doi:10.3389/fonc.2026.1909255)
Supplement: Supplementary file 3 [file Table2.docx]

**Supplementary Table 2** **|** Summary of covariates adjusted in multivariable Cox regression models across included studies

| Study | Covariates Included in Multivariable Model |
| --- | --- |
| Grossmann 2022 | Age, Gender, Clinical tumor stage, Positive surgical margins, Lymphovascular invasion, Concomitant Carcinoma in situ, Lymph node involvement, Adjuvant chemotherapy |
| Tang 2020 | Age, TNM stage, Tumor grade, NLR, dNLR, PLR, LMR |
| Russo 2023 | Age, Smoking status, Gender, Pathological tumor stage, Lymphovascular invasion, Lymph node invasion, Adjuvant chemotherapy |
| Yilmaz 2020 | Age, Gender, ECOG PS, Systemic chemotherapy, Neoadjuvant chemotherapy, TNM stage, Nodal disease, Lymphovascular invasion, Perineural invasion, Tumor grade |
| Salari 2024 | Age, Neoadjuvant chemotherapy, Pathologic T stage, Lymph node metastasis, Lymphovascular invasion, Tumor grade, NLR, PLR, dNLR |
| Zhang 2022 | Gender, Age, BMI, Smoking history, Hypertension, Diabetes mellitus, Tumor diameter, Tumor number, Tumor grade, pT stage, pN stage, Concomitant CIS, Variant histology, Positive surgical margins, Peripheral nerve invasion, Lymphovascular invasion, Adjuvant therapy |
| Zhang 2019 | TNM stage, Tumor size, Tumor margin, Vessel invasion, CAR, NLR, PLR |
| Yamashita 2021 | Age, Gender, TNM stage, NLR, PLR |
| Yilmaz 2023 | Age, Gender, Pathological T stage, Positive lymph node, Positive surgical margin, Variant histology, Adjuvant chemotherapy |

Abbreviations: BMI, body mass index; CAR, C-reactive protein-to-albumin ratio; CIS, carcinoma in situ; dNLR, derived neutrophil-to-lymphocyte ratio; ECOG PS, Eastern Cooperative Oncology Group performance status; LMR, lymphocyte-to-monocyte ratio; LVI, lymphovascular invasion; NLR, neutrophil-to-lymphocyte ratio; PLR, platelet-to-lymphocyte ratio; PNI, perineural invasion; TNM, tumor-node-metastasis.
