# Supplementary material for: Prognostic value of preoperative systemic immune-inflammation index in patients with bladder cancer undergoing radical cystectomy: a systematic review and meta-analysis
Source: Front Oncol. 2026 Aug 14;16:1909255. doi: 10.3389/fonc.2026.1909255 (PMC13521783; doi:10.3389/fonc.2026.1909255)
Supplement: Supplementary file 4 [file Table3.docx]

**Supplementary Table 3** **|** SII Cutoff values and determination methods across included studies

| Study | SII Cutoff | Determination Method |
| --- | --- | --- |
| Grossmann 2022 | 610 | ROC analysis |
| Tang 2020 | OS547.30 / RFS778 | ROC analysis |
| Russo 2023 | 640.27 | ROC analysis |
| Yilmaz 2020 | 768 | ROC analysis |
| Salari 2024 | 410.66 | ROC analysis |
| Zhang 2022 | 554.23 | ROC analysis |
| Zhang 2019 | 507 | X-tile |
| Yamashita 2021 | 438 | ROC analysis |
| Yilmaz 2023 | 1228 | X-tile |
