# Supplementary material for: Prognostic value of preoperative systemic immune-inflammation index in patients with bladder cancer undergoing radical cystectomy: a systematic review and meta-analysis
Source: Front Oncol. 2026 Aug 14;16:1909255. doi: 10.3389/fonc.2026.1909255 (PMC13521783; doi:10.3389/fonc.2026.1909255)
Supplement: Supplementary file 5 [file Table4.docx]

**Supplementary Table 4** **|** Meta-regression analysis exploring potential sources of heterogeneity in overall survival

| Variable | Regression coefficient (β) | Standard error | 95% CI | p value |
| --- | --- | --- | --- | --- |
| SII cutoff | −0.00082 | 0.00053 | −0.00186 to 0.00022 | 0.124 |
| Median age | 0.01590 | 0.03090 | −0.04460 to 0.07640 | 0.606 |
| Sample size | −0.00011 | 0.00010 | −0.00030 to 0.00009 | 0.281 |
